# Spatial-temporal evolution of population aging in the yangtze river delta urban agglomeration of China

**DOI:** 10.1371/journal.pone.0298199

**Published:** 2024-02-15

**Authors:** Lei Zhang, Jie Tang, Meisa Xu, Daliang Zhang, Haixiao Chen, Dayong Zhang

**Affiliations:** 1 Taizhou Hospital of Zhejiang Province, Zhejiang University, Taizhou, China; 2 School of Economy and Management, Zhejiang Sci-Tech University, Hangzhou, China; 3 School of Management, Zhejiang University, Hangzhou, China; 4 School of Medicine, Hangzhou City University, Hangzhou, China; Shandong University, CHINA

## Abstract

The Yangtze River Delta urban agglomeration (YRDUA) is China’s most representative region with remarkable economic development vitality. The purpose of this study is to provide valuable data analysis to actively respond to the population aging in China. We mainly focus on the spatial and temporal evolution of population aging in YRDUA from 2000 to 2020 using city-level population data. This study constructs a multi-dimensional index system to measure population aging including population aging degree, speed, and density. It finds out: (1) the elderly population rate (EPR), the elder-child ratio (ECR), and the elderly dependency ratio (EDR) in the YRDUA area are gradually increasing from 2000 to 2020. In addition, the trends of these indicators in various cities and regions are relatively consistent. All 27 cities in YRDUA entered an aging society, from the primary to the moderate aging stage from 2000 to 2010 and from the moderate to the hyper aging stage from 2010 to 2020. (2) the absolute and relative growth rate of EPR is increasing from 2000 to 2020. However, the absolute and relative growth rate of ECR is increasing from 2000 to 2010 and then decreasing from 2010 to 2020. These results indicate that the two-child policy adopted by the Chinese government plays a positive role. (3) the density level of the elderly population in the YRDUA evolved from low in 2000 to middle in 2010 and then to high in 2020. (4) There are remarkable differences in the process of population aging among three provinces and one city. The contribution of this study is mainly reflected in two aspects: firstly, it constructs a multi-dimensional index system to measure population aging; secondly, using this multi-dimensional index system, it systematically observes the spatial and temporal evolution of population aging from 2000 to 2020 in the Yangtze River Delta Urban Agglomeration.

## Introduction

The elderly population is increasing in number and proportion of the total population, which is becoming one of the most severe demographic challenges globally [1,2]. With the increased ratio and size of the elderly population, China is one of the world’s fastest-aging countries [3,4]. Considerable research considered population aging as a heavy burden on social and economic systems [5]. As pointed out in the *Research Report on the Forecast of Population Aging Development Trend in China* [6], from 2001 to 2100, the development trend of population aging in China can be divided into the rapid aging stage from 2001 to 2020, the accelerated aging stage from 2021 to 2050, and the stable severe aging stage from 2051 to 2100. China presents the typical characteristics of “getting old before getting rich” [7] and “getting old before being prepared” [8]. However, the rapid increase in the degree of population aging in China is accompanied by unbalanced regional distribution and noticeable intra-regional differences [9], and there is a higher concentration of the aging population in the eastern part of China [10].

The Yangtze River Delta urban agglomeration is one of the regions with the most dynamic economy, the most vital ability of innovation, and the most prominent absorption of foreign population in the eastern part of China [11–13], plays an essential strategic role in the overall situation of national modernization and the all-around opening-up pattern [14]. The Yangtze River Delta is one of the areas with a high risk of severe aging in China [15], the degree of population aging increased from 2000 to 2010 [16]. Compared with the Beijing–Tianjin–Hebei and the Pearl River Delta urban agglomerations, intraregional differences in population aging in the Yangtze River Delta are manifested and develop faster [17,18]. Meanwhile, the Yangtze River Delta urban agglomeration has the most healthy and rich elderly population in China [19,20], these old people are likely to be converted into population resources [21]. The Yangtze River Delta urban agglomeration is a pilot area for the Chinese government’s policies, such as the ongoing construction of the Common Prosperity Demonstration Zone in Zhejiang Province, which is more likely to explore effective solutions and methods to cope with the aging population.

The objective of this study is to find out the spatial and temporal population aging evolution in the most remarkable region in China. This study mainly focuses on the spatial and temporal evolution of population aging in the Yangtze River Delta urban agglomeration from 2000 to 2020 using city-level data. Compared with previous studies, this study constructs a multi-dimensional index system to measure population aging. Meanwhile, other studies mainly focused on the data of China and the Yangtze River Delta urban agglomerations in 2000 and 2010 [14], we use the updated data of 2000, 2010, and 2020 with a longer time period to study the changes of population aging in the Yangtze River Delta urban agglomeration. The purpose of this study is to provide valuable data analysis to actively respond to the population aging in China.

## Method and materials

### Study area and data sources

Mainland of China consists of 22 provinces, 5 autonomous regions, and 4 municipalities directly under the central government [22], including 286 cities [23]. The Yangtze River Delta urban agglomeration includes 27 cities from three provinces, which are Jiangsu, Zhejiang, and Anhui provinces, including Shanghai, which is a municipality directly under the central government [24]. The Yangtze River Delta urban agglomeration has abundant resources and large market potential and is an important industrial and commercial center in China [12]. As proposed in the *Plan of Yangtze River Delta Urban Agglomeration Development* (2016), a world-class urban agglomeration with a global influence in an all-around way should be built. Furthermore, the *Three-year Action Plan for the Integrated Development of the Yangtze River Delta* (2018–2020) clarified the tasks, time, and route of the integrated development of the Yangtze River Delta. The Yangtze River Delta consists of 27 cities, namely, Nanjing, Wuxi, Changzhou, Suzhou, Nantong, Yangzhou, Zhenjiang, Yancheng, and Taizhou cities in Jiangsu Province; Hangzhou, Ningbo, Wenzhou, Huzhou, Jiaxing, Shaoxing, Jinhua, Zhoushan, and Thaizhou cities in Zhejiang Province; and Hefei, Wuhu, Maanshan, Tongling, Anqing, Chuzhou, Chizhou, and Xuancheng cities in Anhui Province and Shanghai municipality.

Through data collection and aggregation, the data from the fifth [25], sixth [26], and seventh [27] National Population Censuses of China and the statistical yearbook of Shanghai City [28–30], Jiangsu [31–33], Anhui [34–36], and Zhejiang provinces [37–39] were incorporated into our research data, the aging progress and present situation of the Yangtze River Delta urban agglomeration and even each city were explored. The spatial-temporal differences in population aging within the Yangtze River Delta urban agglomeration were analyzed from three levels—the degree, speed, and density of population aging.

### Population aging indicator

The United Nations defines population aging as a country or region where the population aged 60 and over exceeds 10% or the population aged 65 and over exceeds 7%. Table 1 presents the criteria for defining the types of the population age structure in *Aging and Its Socioeconomic Impacts* published by the United Nations in 1956 [40] and at the first World Assembly on Ageing convened by the United Nations in 1982. Based on the proportion of the elderly population in major European Union countries, regional population aging had become one of the common problems in population development [41]. Some essential indexes include the total population, age structure, and dependency ratio to measure and analyze the population [42]. The Theil index analysis method is used to analyze the regional differences and influencing factors of China’s population aging by selecting two indexes: the aging coefficient and the elderly-to-child ratio [43].

**Table 1:** Population age structure.

	Young	Adult	Elder
Elderly population rate (EPR)	Below 4%	4%-7%	Above 7%
Elder-child ratio (ECR)	Below 15%	15%-30%	Above 30%

In this study, the analysis was performed using the elderly population rate (EPR), the elder-child ratio (ECR), and the elderly dependency ratio (EDR). The specific index meanings are displayed below

### EPR

In a certain period of time, the proportion of the elderly population in the total population in a specific area. The most commonly used and representative index reflects the proportion of elderly population and the aging degree [2]. EPR is the elderly population rate, EP denotes the number of the elderly people over 65 years old, and P represents the total population.


$$EPR=\frac{EP}{P}=\frac{\sum_{i=65}^{\omega }X_{i}}{\sum_{i=0}^{\omega }X_{i}}$$ (1)

### ECR

ECR refers to the ratio of the number of elderly aged 65 and above to the number of children aged 0–14, which is commonly expressed as a percentage and is an important index reflecting the age structure and aging degree of a country or region [44]. EP is the number of the elderly population over 65 years old, and C represents the number of children aged 0–14 years old. When ECR ≧ 30%, the region enters an aging society.


$$ECR=\frac{EP}{C}=\frac{\sum_{i=65}^{\omega }X_{i}}{\sum_{i=0}^{14}X_{i}}$$ (2)

### EDR

EDR refers to the ratio of the middle-aged and old-aged part of the non-working-age population to the working-age population. EDR is also one of the indexes reflecting the social consequences of population aging from an economic perspective [2]. EDR stands for the dependency ratio of the elderly population, EP is the number of the elderly population over 65 years old, and W denotes the number of labor force aged 15–64 years old.


$$EDR=\frac{EP}{W}=\frac{\sum_{i=65}^{\omega }X_{i}}{\sum_{i=15}^{64}X_{i}}$$ (3)

*i* represents age, ω represents maxmium age, *Xi* means the population with *i* age.

### Measurement of population aging

Based on a thorough review, synthesis, and understanding of the existing studies, we constructed a multi-dimensional index system to measure population aging. The degree, speed, and density of population aging are used to measure the population aging in the Yangtze River Delta.

### Degree of population aging

The degree of population aging is the common index to measure population aging. This part uses the EPR, ECR, and EDR to measure the degree of population aging.

### Speed of population aging

Based on the methods of Wang et al. [45], this study measures the speed of population aging with the absolute growth rate (AGR) and relative growth rate (RGR) of population aging indexes. As an example of the EPR, the AGR of EPR from 2000 to 2010 is the data of EPR in 2010 minus the data of EPR in 2000. RGR of EPR from 2000 to 2010 is the data of AGR of EPR from 2000 to 2010 divided by the data of EPR in 2000, where *i* represents city, and *t* represents time. The calculation is the same for the speed of the ECR.


$$AGR_{EPR_{i(t-1∼t)}}=EPR_{it}-EPR_{i(t-1)}$$ (4)

$$RGR_{EPR_{i(t-1∼t)}}=\frac{EPR_{it}-EPR_{i(t-1)}}{EPR_{i(t-1)}}$$ (5)

### Density of population aging

With reference to the calculation formula of population density = population size (person)/area (square kilometer), the density of the elderly population = the size of the elderly population over 65 years (person)/area (square kilometer).

## Results

Three dimensions of indexes, namely, degree, speed, and density, are used to measure the population aging in the Yangtze River Delta urban agglomeration.

### Spatial-temporal evolution analysis of population aging degree in the Yangtze River Delta

**Fig 1:**
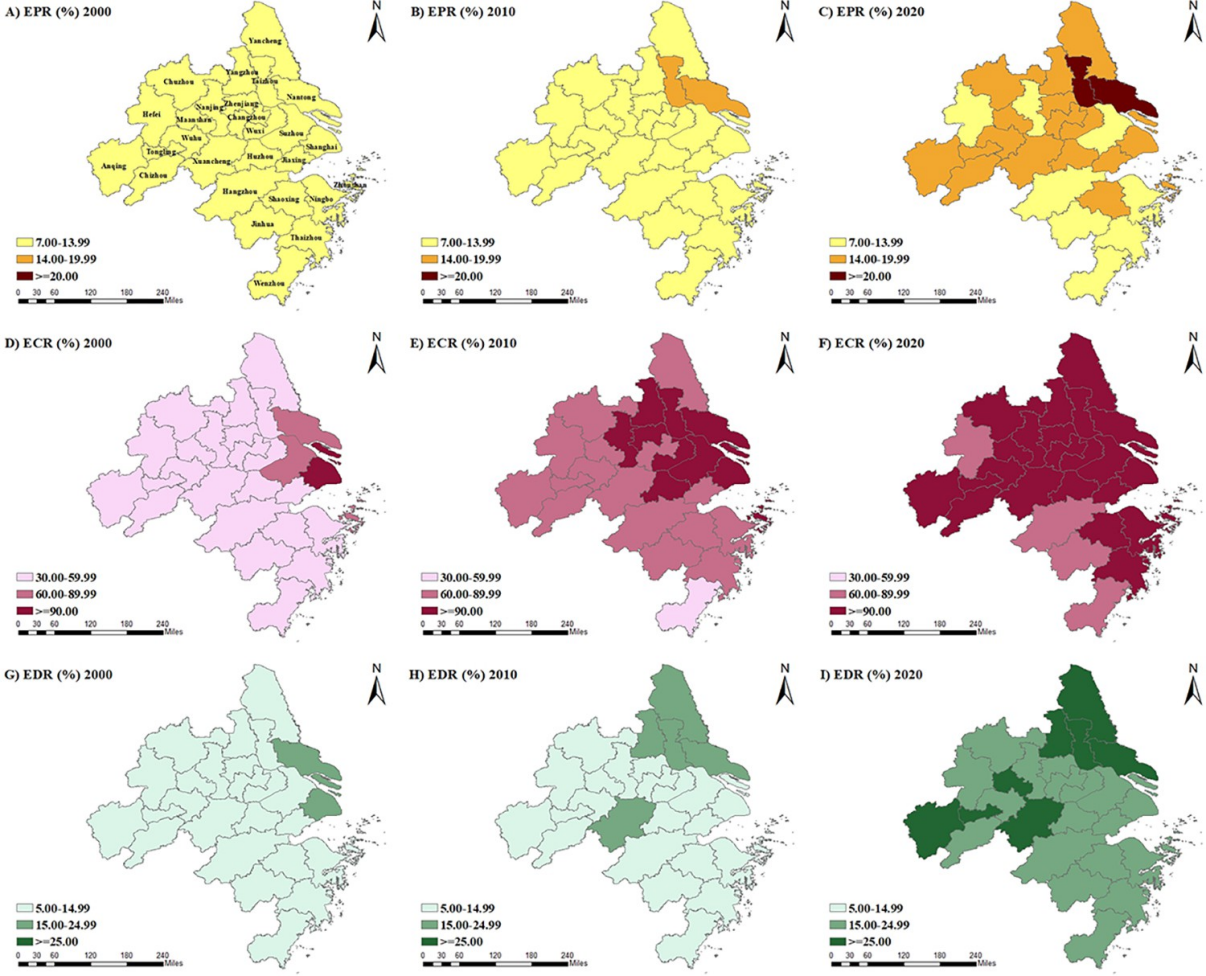
Population aging degree of Yangtze River Delta urban agglomeration. (Shape file source: Republished from http://www.gscloud.cn under a CC BY license, with permission from Geospatial Data Cloud, original copyright [2022]; The map was created using ArcGIS 10.2 software and the calculation data of EPR, ECR, and EDR is sourced from relevant statistical yearbook).

SPSS software is used to calculate the original data according to the Formulas (1)(2)(3), three index values (EPR, ECR, and EDR) of the Yangtze River Delta urban agglomeration in 2000, 2010, and 2020 were obtained, and ArcGIS software is used to draw the map, as shown in Table 2 and Fig 1.

**Table 2:** Population aging degree of Yangtze River Delta Urban Agglomeration in 2000, 2010 and 2020. Unit: %.

Cities	2000			2010			2020		
	EPR	ECR	EDR	EPR	ECR	EDR	EPR	ECR	EDR
Shanghai	11.46	93.52	15.02	10.13	117.65	12.47	16.28	166.20	22.02
Nanjing	8.49	55.02	11.16	9.20	96.67	11.31	13.7	107.43	18.62
Wuxi	8.77	54.14	11.69	9.50	92.24	11.84	14.65	113.08	20.25
Changzhou	8.75	52.77	11.72	9.78	84.95	12.42	14.88	112.24	20.71
Suzhou	9.58	66.48	12.60	8.51	92.47	10.34	12.44	91.81	16.81
Nantong	12.44	71.87	17.71	16.51	155.36	22.66	22.67	208.01	34.12
Yancheng	7.93	39.93	10.98	11.97	83.40	16.25	19.88	132.24	30.54
Yangzhou	9.14	52.95	12.42	12.45	105.35	16.44	19.99	172.86	29.21
Zhenjiang	8.74	52.40	11.72	10.36	100.61	13.06	17.51	147.31	24.81
Taizhou	10.30	54.58	14.54	14.24	117.63	19.34	22.01	178.07	33.53
Hangzhou	8.83	53.68	11.82	9.02	79.19	11.33	11.66	89.56	15.48
Ningbo	8.75	54.11	11.65	8.61	73.65	10.80	12.59	102.67	16.76
Wenzhou	7.31	36.90	10.02	7.62	53.28	9.76	11.71	76.66	16.04
Jiaxing	9.59	53.88	13.21	10.89	83.74	12.79	14.05	116.27	19.01
Huzhou	9.94	53.73	13.89	10.89	93.32	14.06	15.52	129.98	21.39
Shaoxing	9.69	52.61	13.48	9.94	77.78	12.86	16.21	136.30	22.54
Jinhua	8.81	48.09	12.09	9.10	63.95	11.87	11.59	81.02	15.64
Zhoushan	9.33	61.06	12.37	10.50	103.04	13.24	17.09	174.17	23.38
Thaizhou	9.05	50.11	12.42	9.83	63.58	13.16	13.80	93.94	19.30
Hefei	6.95	30.95	9.85	8.46	60.21	10.92	11.99	72.58	16.77
Wuhu	7.48	35.37	10.49	10.76	85.60	14.03	16.11	110.80	23.23
Maanshan	7.4	33.9	10.45	10.63	83.18	13.88	17.53	122.16	25.74
Tongling	6.69	32.94	9.17	10.15	74.17	13.33	17.46	121.50	25.61
Anqing	7.41	26.93	11.38	10.34	62.55	14.14	17.06	102.28	25.75
Chuzhou	7.76	29.48	11.76	10.75	62.50	14.92	15.97	98.22	23.57
Chizhou	7.72	32.49	11.28	10.03	61.46	13.62	16.74	107.45	24.73
Xuancheng	7.78	37.66	10.86	11.44	78.52	15.46	18.04	132.26	26.41

Note: The data in the table are sourced from the 5th, 6th, and 7th National Population Censuses of China, and the statistical yearbooks of Anhui Province, Jiangsu Province, Zhejiang Province and Shanghai City.

Comparing the standard of population age structure in Tables 1 and 2, the EPR of all cities except Tongling and Hefei was ≧7% in 2000, and the EPR of 27 cities in the Yangtze River Delta in 2010 and 2020 was greater than 7%. Moreover, the elderly level increased evidently. The ECR in all other 24 cities except Anqing City and Chuzhou City was above 30%, entering the old age structure in 2000. As a whole, cities in the Yangtze River Delta urban agglomeration presented an elder age structure in 2000, and by 2020, the age ratio of all cities further increased.

The three stages of society are classified based on the proportion of the elderly population of the entire population in a country [46]. The first aging stage refers to countries with 7%–14% of people aged 65 and above. The next stage is when the elderly population dominates the country by 14%–20%, whereas the last stage of hyper-aged has a population of 65 years and above 20% or more, as shown in Table 3.

**Table 3:** Stages of population aging degree.

	Primary Aging Stage	Moderate Aging Stage	Hyper Aging Stage
Elderly population rate (EPR)	7%≦A<14%	14%≦A<20%	A≧20%
Elder-child ratio (ECR)	30%≦B<60%	60%≦B<90%	B≧90%

According to the classification criteria of population aging stages (Table 3), the aging degree gradually deepened from 2000 to 2020. Based on the EPR, in 2000, all 27 cities were in the primary aging stage. In 2010, Nantong and Taizhou entered the moderate aging stage, whereas the other 25 cities still kept the primary aging stage. By 2020, Nantong and Taizhou stepped into the hyper-aging stage. Then, 18 cities, namely, Shanghai, Yancheng, Wuxi, Changzhou, Yancheng, Yangzhou, Zhenjiang, Jiaxing, Huzhou, Shaoxing, Zhoushan, Wuhu, Maanshan, Tongling, Anqing, Chuzhou, Chizhou, and Xuancheng stepped into the moderate aging stage. Moreover, eight cities, that is, Nanjing, Suzhou, Hangzhou, Ningbo, Wenzhou, Jinhua, Thaizhou, and Hefei, keep the primary aging stage.

In terms of the ECR, in 2000, Shanghai entered a hyper-aging stage; Suzhou, Nantong, and Zhoushan entered a moderate aging society; and other cities were all in the primary aging stage. In 2010, a total of 10 cities, namely, Shanghai, Nanjing, Wuxi, Suzhou, Nantong, Yangzhou, Zhenjiang, Taizhou, Huzhou, and Zhoushan, experienced hyper aging stag. Moreover, 16 cities, namely, Changzhou, Yancheng, Hangzhou, Ningbo, Jiaxing, Shaoxing, Jinhua, Thaizhou, Hefei, Wuhu, Maanshan, Tongling, Anqing, Chuzhou, Chizhou, and Xuancheng, entered the moderate aging stage. Meanwhile, Wenzhou remained in the primary aging stage. In 2020, 23 cities in the Yangtze River Delta underwent the hyper-aging stage. Only Hangzhou, Wenzhou, Jinhua, and Hefei were in the moderate aging stage.

In terms of the EDR, measuring the burden of the elderly population is an important indicator. From 2000 to 2020, the average EDR of 27 cities in the Yangtze River Delta urban agglomeration increased gradually, from approximately 10% in 2000 to approximately 20% in 2020. Therefore, in 2000, 10 working people supported one person aged 65 or older, and in 2020, five working people supported one person aged 65 or older. Thus, the elderly population burden is rising rapidly. In 2020, the ERD value of Nantong, Yancheng, and Taizhou was above 30%, ranking the top three. In general, the EDR level of Shanghai, Jiangsu Province, and Anhui Province is high, whereas that of Zhejiang Province is low.

On the whole, the measurement results of the three indexes, namely, the EPR, ECR, and EDR, were relatively consistent. All 27 cities in the Yangtze River Delta urban agglomeration entered an aging society. From 2000 to 2010, they developed from the primary aging stage to the moderate aging stage, and from 2010 to 2020, they transformed from the moderate aging stage to the hyper-aging stage. Thus, the aging degree gradually deepened. The aging level of Nantong was the highest in three years, and the aging level of Shanghai, Yangzhou, Taizhou, and Zhoushan was relatively high.

### Spatial-temporal evolution analysis of population aging speed in the Yangtze River Delta

The AGR and RGR of the EPR and ECR in the Yangtze River Delta urban agglomeration in two time periods—2000–2010 and 2010–2020—were quantitatively analyzed according to Formulas (4)(5), with the obtained data listed in Table 4.

**Table 4:** Speed of population aging in Yangtze river delta urban agglomeration (2000–2010). Unit: %.

Cities	elderly population rate (EPR)				elder-child ratio (ECR)			
	2000–2010		2010–2020		2000–2010		2010–2020	
	Absolute growth rate	Relative growth rate	Absolute growth rate	Relative growth rate	Absolute growth rate	Relative growth rate	Absolute growth rate	Relative growth rate
Shanghai	-1.33	-11.61	6.15	60.71	24.06	25.72	48.55	41.27
Nanjing	0.69	8.13	4.52	49.24	41.51	75.44	10.9	11.29
Wuxi	0.71	8.10	5.17	54.54	37.90	70.01	21.04	22.86
Changzhou	1.02	11.66	5.11	52.30	32.11	60.84	27.36	32.23
Suzhou	-1.08	-11.27	3.94	46.35	25.81	38.82	-0.48	-0.52
Nantong	4.06	32.64	6.17	37.39	83.36	115.99	52.79	34.01
Yancheng	4.03	50.82	7.92	66.22	43.36	108.58	48.95	58.77
Yangzhou	3.30	36.11	7.55	60.69	52.29	98.75	67.61	64.24
Zhenjiang	1.61	18.42	7.16	69.18	48.09	91.77	46.82	46.59
Taizhou	3.93	38.16	7.78	54.67	62.92	115.28	60.56	51.54
Hangzhou	0.19	2.15	2.64	29.27	25.51	47.53	10.37	13.10
Ningbo	-0.14	-1.60	3.98	46.23	19.54	36.11	29.02	39.40
Wenzhou	0.31	4.24	4.09	53.67	16.38	44.39	23.38	43.88
Jiaxing	0.40	4.17	3.16	29.02	29.86	55.43	32.53	38.85
Huzhou	0.95	9.56	4.63	42.52	39.59	73.68	36.66	39.28
Shaoxing	0.25	2.58	6.27	63.08	25.17	47.85	58.52	75.24
Jinhua	0.29	3.29	2.49	27.36	15.86	32.98	17.07	26.69
Zhoushan	1.17	12.54	6.59	62.76	41.98	68.76	71.13	69.03
Thaizhou	0.78	8.62	3.97	40.39	13.47	26.89	30.36	47.75
Hefei	1.51	21.73	3.53	41.73	29.26	94.54	12.37	20.54
Wuhu	3.28	43.85	5.35	49.72	50.23	142.01	25.2	29.44
Maanshan	3.23	43.65	6.9	64.91	49.28	145.37	38.98	46.86
Tongling	3.46	51.72	7.31	72.02	41.23	125.17	47.33	63.81
Anqing	2.93	39.54	6.72	64.99	35.62	132.27	39.73	63.52
Chuzhou	2.99	38.53	5.22	48.56	33.02	112.01	35.72	57.15
Chizhou	2.31	29.92	6.71	66.90	28.97	89.17	45.99	74.83
Xuancheng	3.66	47.04	6.6	57.69	40.86	108.50	53.74	68.44

Note: The data in the table are sourced from the 5th, 6th, and 7th National Population Censuses of China, and the statistical yearbooks of Anhui Province, Jiangsu Province, Zhejiang Province and Shanghai City.

Based on the AGR and RGR of the EPR, the growth rate in the decade from 2000 to 2010 was slow, where the AGR was approximately 1%, the RGR was approximately 10%, and the highest RGR was 50%. The EPR of the three cities showed negative growth. However, the AGR and RGR were very fast in the decade from 2010 to 2020. In this period, the AGR of most cities in the Yangtze River Delta urban agglomeration was over 5%, and the RGR was approximately 50%, 72% at maximum. From the geographical distribution perspective, the EPR in Shanghai exhibited a negative growth from 2000 to 2010 and increased significantly from 2010 to 2020, with an RGR of 60%. The period 2000–2010 witnessed a positive growth of the EPR in all cities except Shanghai, Suzhou, and Ningbo. The fastest RGR appeared in Tongling, Yancheng and Xuancheng. In 2010–2020, the EPR of all cities was growing rapidly and was the highest in Tiongling, Zhenjiang, Chizhou, and Yancheng, with 72.02%, 69.18%, 66.90%, and 66.22%, respectively.

As for the AGR and RGRs of the ECR, the AGR was within 50%, and the RGR was around 100% during the decade from 2000 to 2010. The fastest RGR appeared in three cities, Maanshan, Wuhu, and Anqing, with 145.37%, 142.01%, and 132.27%, respectively. Nonetheless, during 2010–2020, the AGR of this index was accelerated somehow, whereas the RGR was slowed down. The fastest RGR of the ECR appeared in Shaoxing, Chizhou, Zhoushan, and Xuancheng, being 75.24%, 74.83%, 69.03%, and 68.44%, respectively.

On the whole, the AGR and RGR of the EPR are increasing from 2000 to 2020, whereas the AGR and RGR of the ECR are increasing from 2000 to 2010, then decreased from 2010 to 2020. The growth rate of population aging in Shanghai and Zhejiang Province was relatively slow, whereas that in cities of Anhui Province and Jiangsu Province was relatively fast, manifesting specific differences among the three provinces and one city (Jiangsu Province, Zhejiang Province, Anhui Province, and Shanghai City) in the population aging speed.

### Spatial-temporal evolution analysis of population aging density in the Yangtze River Delta

The results of population aging density in the Yangtze River Delta urban agglomeration is showed in Table 5.

**Table 5:** Density of population aging in Yangtze River delta urban agglomeration. Unit: Person/km^2^.

Cities	Density of the elderly population in 2000	Density of the elderly population in 2010	Density of the elderly population in 2020
Shanghai	302	368	639
Nanjing	80	112	194
Wuxi	98	131	236
Changzhou	77	103	180
Suzhou	78	105	183
Nantong	119	150	166
Yancheng	43	51	79
Yangzhou	64	84	138
Zhenjiang	66	84	146
Taizhou	87	114	172
Hangzhou	37	47	83
Ningbo	56	67	121
Wenzhou	47	59	92
Jiaxing	88	125	180
Huzhou	45	54	90
Shaoxing	50	59	103
Jinhua	37	45	75
Zhoushan	65	82	136
Thaizhou	50	62	91
Hefei	42	68	98
Wuhu	49	73	98
Maanshan	52	86	94
Tongling	41	66	76
Anqing	29	36	52
Chuzhou	24	31	47
Chizhou	14	17	27
Xuancheng	17	24	37

Note: The data in the table are sourced from the 5th, 6th, and 7th National Population Censuses of China, and the statistical yearbooks of Anhui Province, Jiangsu Province, Zhejiang Province and Shanghai City.

With Tables 5 and 6 combined, from 2000 to 2010 and then to 2020, the density level of the elderly population in the Yangtze River Delta urban agglomeration evolved from low to middle and then to high. In 2000, 13 cities in the Yangtze River Delta urban agglomeration were in the low-density area of the elderly population, 12 cities were in the middle-density area of the elderly population, one city was in the upper-middle-density area of the elderly population, and one city was the high-density area. Most cities in the Yangtze River Delta urban agglomeration were in the medium-low density area of the elderly population in 2000. By 2010, six cities in the Yangtze River Delta urban agglomeration were in the low-density area of the elderly population, 13 cities were in the middle-density area of the elderly population, six cities were in the upper-middle-density area of the elderly population, and two cities in were the high-density area. By 2020, three cities in the Yangtze River Delta urban agglomeration were in the low-density area of the elderly population, 11 cities were in the middle-density area of the elderly population, five cities were in the upper-middle-density area of the elderly population, and eight cities were in the high-density area of the elderly population. In 2020, many cities of the Yangtze River Delta urban agglomeration were in the middle-high density of the elderly population.

**Table 6:** Standard for density of population aging. Unit: Person/km^2^.

	Low density of the elderly population	Middle density of the elderly population	Upper middle density of the elderly population	High density of the elderly population
(D)	D<50	50≦D<100	100≦D<150	D≧150

From the spatial distribution perspective, the density of population aging of cities in the Yangtze River Delta urban agglomeration also showed spatial differences. Significant differences among the three provinces and one city showed an outward diffusion trend from the center. The density of the elderly population in Shanghai had been high, reaching 302 people/km^2^ in 2000, and increased rapidly in the past two decades, reaching 639 people/km^2^ in 2020. The density of the elderly population in Jiangsu Province ranked second overall, particularly in Nantong City, which entered a high-density area of the elderly population in 2010. Wuxi, Nanjing, Suzhou, Changzhou, Taizhou, and Nantong were all high-density areas of the elderly population in 2020. The population density of the elderly in Zhejiang Province ranked third in the Yangtze River Delta urban agglomeration. By 2020, except Jiaxing, Zhoushan, Ningbo, and Shaoxing, other cities were middle-density areas of the elderly population. The overall density of the elderly population in Anhui Province was not high, all being in the middle and low-density areas.

## Discussion

This study illustrated the marked spatial-temporal evolution of population aging of degree, speed, and density in the Yangtze River Delta urban agglomeration from 2000 to 2020.

First, the analysis of population aging degree 2000–2010 witnessed the development of the Yangtze River Delta urban agglomeration from the primary aging stage to the moderate aging stage, whereas 2010–2020 witnessed its development from the moderate aging stage to the hyper aging stage. The EPR, ECR, and EDR in Shanghai, Nantong, Yangzhou, Taizhou, and Zhoushan were much higher than those in other cities. From the perspective of spatial distribution, Jiangsu province is the most serious degree of population aging among the Yangtze River Delta urban agglomerations, the three indicators including EPR, ECR, and EDR in Jiangsu province are relatively more serious from 2000 to 2020 than other regions. Anhui province has the second most serious degree of population aging in three indicators including EPR, ECR, and EDR. The ECR index of Shanghai is relatively serious level, while the other indexes EPR and EDR are relatively light. The degree of population aging in Zhejiang Province including EPR, ECR, and EDR is relatively light in the Yangtze River Delta urban agglomeration. Similar to our results, Xu et al. found that there was a higher level of population aging in the Nantong–Taizhou–Yangzhou agglomeration of Jiangsu Province and southwestern Zhejiang Province from 2000 to 2010 [47]. Liu NRS et al. believed that the level of population aging in China continued to deepen, whereas the regional gap continued to widen, and the degree of aging remained too high in some provinces and regions [48].

Second, the analysis of population aging speed showed that the AGR and RGR of the EPR and the ECR were opposite from 2000 to 2020. The AGR and RGRs of the EPR are higher from 2010 to 2020 than those from 2000 to 2010. From the spatial distribution perspective, the growth rate of population aging in Shanghai and Zhejiang Province was relatively slow, whereas that in the cities of Anhui Province and Jiangsu Province was relatively fast. Particularly, Tongling, Zhenjiang, and Chizhou were evidently faster than in other cities in the Yangtze River Delta urban agglomeration. The AGR and RGRs of the ECR are lower from 2010 to 2020 than those from 2000 to 2010. This case is probably related to China’s relaxation of the two-child policy, which implemented the two-child fertility policy for couples where either is from a single-child family in 2011, two-child fertility policy for couples where either the husband or the wife is from a single-child family in 2013, and the universal two-child policy in 2015. The implementation of the two-child policy played a role in increasing the birth population and decreasing the rapid population aging [49,50]. Therefore, the two-child policy effectively reduced the growth level of the ECR. Particularly, in Maanshan, Wuhu, and Anqing cities, the AGR and RGRs of the ECR decreased sharply.

Third, the density level of the elderly population in the Yangtze River Delta urban agglomeration was developed from low- to middle-density areas in the decade from 2000 to 2010 and evolved from middle- to high-density areas in the decade from 2010 to 2020. From the spatial distribution perspective, the density of the elderly population in Shanghai had been high, The density of the elderly population in Jiangsu Province ranked second, the population density of the elderly population in Zhejiang Province ranked third. The overall density of the elderly population in Anhui Province was not high, all being in the middle and low-density areas. The density of the elderly population in Shanghai, Wuxi, and Nanjing was much higher than that in other cities in the Yangtze River Delta urban agglomeration, these cities have more wealthy elderly people.

The spatial and temporal evolution of population aging in the Yangtze River Delta urban agglomeration may be attributed to several possible reasons: First, from regional perspective, the Yangtze River Delta urban agglomeration is among the areas in China with a higher level of population aging. This may be due to the region having a better healthcare system as a result of its economic development, leading to a higher proportion of the aging population; Second, within the Yangtze River Delta urban agglomeration, different cities exhibit varying degrees and rates of progression in population aging. Some cities with lower economic levels, like Nantong in Jiangsu Province, have higher levels of population aging. The reason may be that labor force migration is convenient, it is easier to migrate from lower economic cities to neighboring cities with higher economic development level.

Healthy elderly population is probably a human resource for the city [21,51]. Recent research found that the eastern coastal of China have a higher coupling and coordination degree between the elderly population ratio and per capita Gross Regional Product (GRP) compared to the central and western regions, indicating a greater capacity in the eastern regions to effectively respond to the challenges of population aging [52]; Therefore, we think that the aging population in the Yangtze River Delta region can be a resource. The elderly population brings a wealth of experience, knowledge, and skills that can benefit society. They can contribute to the workforce through part-time or volunteer work, and their pensions and savings can stimulate the economy [53,54]. The key to ensuring that the aging population is a resource rather than a burden is implementing policies that promote healthy aging and provide adequate support to the elderly population. The elderly labor resources should be fully utilized, and healthy aging should be promoted. With the faster development of the economy, compared with the elderly population throughout China, the elderly groups in the Yangtze River Delta urban agglomeration show higher physical quality and ideological and spiritual quality [55,56]. Thus, the elderly with spare capacity should be called upon and advocated to extend their retirement age and make full use of the elderly labor resources. Moreover, the values of the elderly should be transformed to promote the re-employment of the elderly and achieve productive old age.

The 27 cities in the Yangtze River Delta urban agglomeration display a high level of urbanization, and the level of urban public services is also relatively in the lead across China. We should establish and improve the medical security system for the elderly population and effectively improve the medical facilities and medical insurance systems to meet the increasing medical needs of the elderly population. The role of community services in facing population aging should be sufficiently exerted, for example, by improving community services for the elderly and establishing a mutual aid system for community residents to improve home-based care. Efforts should also be made to facilitate the construction of socialized old-age service institutions, such as nursing homes, village-level centralized support points, and old-age care centers, thereby improving community endowment.

This study has some limitations. Our results are based on city-level data, and this study does not observe the urban–rural differences in population aging in the Yangtze River Delta. Considerable research on population aging focused on urban–rural differences. Although the urbanization rate of the Yangtze River Delta urban agglomeration is at the highest level in China, a detailed study can comprehensively examine the degree of population aging in the Yangtze River Delta urban agglomeration from urban and rural aspects.

## Conclusions

In the crucial 20 years when the Yangtze River Delta urban agglomeration entered the aging society, this paper presents the overall outlook of population aging changes and micro-changes at the city level in the Yangtze River Delta region from 2000 to 2020, which provides data support for the follow-up research on population aging countermeasures and programs in the Yangtze River Delta urban agglomeration. It finds out: (1) EPR, ECR, and EDR in the YRDUA area are gradually increasing from 2000 to 2020. In addition, the trends of these indicators in various cities and regions are relatively consistent. All 27 cities in YRDUA entered an aging society, from the primary to the moderate aging stage from 2000 to 2010 and from the moderate to the hyper aging stage from 2010 to 2020. (2) the absolute and relative growth rate of EPR is increasing from 2000 to 2020. However, the absolute and relative growth rate of ECR is increasing from 2000 to 2010 and then decreasing from 2010 to 2020. These results indicate that the two-child policy adopted by the Chinese government plays a positive role. (3) the density level of the elderly population in the YRDUA evolved from low in 2000 to middle in 2010 and then to high in 2020. (4) There are remarkable differences in the process of population aging among three provinces and one city.

The Yangtze River Delta urban agglomeration is a region with continuous economic growth and population migration. For further study, a more detailed study of population aging can be conducted at the district or county levels. The analysis should be conducted from the individual level to investigate how many elderly population in this region is richer and healthier, so that it is more likely to transform the so-called population burden into population resources, and more in-depth data at the individual level is needed for analysis.

## Supporting information

S1 Data (XLS)

## Abbreviations

YRDUA: The Yangtze River Delta urban agglomeration
EPR: Elderly population rate
ECR: Elder-child ratio
EDR: Elderly dependency ratio
AGR: Absolute growth rate
RGR: Relative growth rate
